# Opioid use, motivation to quit, and treatment status related to COVID-19: a cross-sectional study

**DOI:** 10.1186/s13104-021-05601-z

**Published:** 2021-05-20

**Authors:** Maria A. Parker, Jon Agley, Zachary W. Adams, Andrea C. Villanti

**Affiliations:** 1 Department of Epidemiology & Biostatistics, Indiana University School of Public Health, Bloomington, IN USA; 2 Prevention Insights, Department of Applied Health Science, Indiana University School of Public Health, Bloomington, IN USA; 3 Indiana University School of Medicine, Indianapolis, IN USA; 4 Vermont Center on Behavior & Health, University of Vermont, Burlington, VT USA

**Keywords:** Opioids, Survey, Motivation, COVID-19, Treatment

## Abstract

**Objective:**

Persons who use opioids may be at elevated risk of harm from the coronavirus disease 2019 (COVID-19) pandemic, yet few data currently exist that can be used to examine this risk. As part of a rapid response survey, this study measured opioid users’ perceptions of risk or harm from COVID-19, as well as potential changes in motivation to quit, frequency of use, and engagement with treatment. Data collected from Amazon’s Mechanical Turk (n = 562) were analyzed.

**Results:**

Participants perceived modest risk elevation from COVID-19 due to their opioid use, and perceived moderate risk to themselves or their community from COVID-19. Since learning about COVID-19, 31.2% reported decreasing their opioid use, and 26.0% reported increased motivation to quit. Thirty-seven percent of participants reported both their use and motivation to quit stayed the same; 16.6% reported decreased use and increased motivation to quit. Participants who reported that their opioid use increased after learning about COVID-19, or whose motivation to quit opioids decreased, were more likely to also be engaged in treatment than those whose use or motivation stayed the same. These preliminary findings suggest that there likely is an association between COVID-19, opioid use, and treatment engagement that merits further in-depth investigation.

## Introduction

Major disasters long have been associated with substantive adverse outcomes, including anxiety and substance use [1]. Similarly, the coronavirus 2019 (COVID-19; Severe Acute Respiratory Syndrome Coronavirus 2 [SARS-CoV-2]) pandemic is likely to pose serious problems for persons with opioid use disorder (OUD) [2]. Although the health consequences of the pandemic remain unclear [3], evidence suggests there have been challenges in maintaining substance use disorder treatment services [4], and a recent content review of COVID-19 and addiction suggests risk of increases in prevalence of withdrawal symptoms and addictive behaviors, including relapse [5].

Persons with substance use disorders, such as OUD, are likely at greater risk of worse COVID-19 outcomes [6, 7]. For example, individuals who use opioids may be more likely than others to experience worsened hypoxemia or be subjected to “triage bias” [8]. Further, individuals with OUD have more co-occurring disorders, an increased prevalence of cigarette smoking [9], and often live in situations where social distancing is not possible [10]. A growing body of literature has also focused on the “collision” of the COVID-19 pandemic and opioid epidemic [10, 11], with attention to persons with psychiatric comorbidities. Finally, there is preliminary evidence of an increase in opioid overdoses during the early months of the pandemic, compared to a pre-COVID period [12, 13].

Amidst the ongoing pandemic, data remain sparse [3], and there has been little research to assess whether COVID-19 has affected opioid use and motivation to stop using opioids. Preliminary studies show some increases in opioid use through drug testing data [14, 15]. However, the issue is complex and will require concerted effort from the field to address. To facilitate rapid response and data collection, the authors were able to collect data as part of a multiple-team, combined questionnaire administration for multiple types of substance use. The goal of this specific component of the study was to explore the relationship between treatment for OUD, changes in opioid use, and changes in motivation to stop using opioids among persons who use opioids, in the context of the COVID-19 pandemic.

## Main text

### Materials and methods

#### Data collection

An online survey using web-based crowdsourcing was conducted via Amazon Mechanical Turk (mTurk) between April 22 and May 17, 2020. The overall survey was designed to examine the relationship between treatment for OUD and cigarette smoking among persons with recent opioid use [17]. For this research note, questions related to COVID-19 were included because of the importance of assessing the impact of the pandemic on persons who use opioids.

Screened participants (n = 1022) were ages 18 and older, were United States residents, had used opioids in their lifetime, and had a Human Intelligence Task (HIT) approval rate greater than 90. ‘HIT’ is the name for the virtual tasks (i.e., question that needs an answer) posted on mTurk that potential respondents can complete and submit in exchange for payment of a predetermined amount of money if the task is completed as intended (i.e., “approved”) (see www.mturk.com for more detail). The analytic subsample for this study comprised persons who endorsed any past-month prescription opioid misuse or heroin use (n = 600) and answered all COVID-related survey questions (n = 562 [94% response rate]) (see “Measures”). While “crowdsourced” data collection using mTurk is relatively new, evidence suggests that it compares favorably to other types of participant pools [18, 19], tends to be reliable and valid [20], and in many ways reflects the overall US population [21], though there are also some differences (e.g., intellectual abilities and level of education) [22]. Using a competing platform that is similar to mTurk, researchers were also able to replicate well-established experimental findings [23]. For this study, in response to recent concerns about non-compliant mTurk workers [24], checks were embedded in the Qualtrics survey to eliminate potential bots, avoid duplicate responses, and ensure eligibility.

#### Measures

##### Sociodemographics

Respondents were asked to indicate their sex [male/female], age, race/ethnicity [White, Black, Hispanic, Other], and education [≤ High School, Some College, Bachelor’s, > 4 years College].

##### Opioid use and treatment

Participants were asked, “Have you ever, even once, used any prescription pain reliever in any way a doctor did not direct you to use it, including using it without a prescription of your own, using it in greater amounts, more often, or longer than you were told to take it, or using it in any other way a doctor did not direct you to use it?” [25, 26]. This was followed by questions that asked how many days in the last month respondents used (1) prescription opioids or (2) heroin. An additional question asked, “Are you currently enrolled in a treatment program for your prescription opioid or heroin use?”.

##### COVID-19 perceptions, opioid use and motivation to quit

Items on COVID-19 were adapted from a recent tobacco study [16]. Participants were asked, “How concerned about coronavirus (COVID-19) are you for your own health?” and “How concerned about coronavirus (COVID-19) are you for the health of others in your community?” both with response options ranging from [0 = Not at all to 7 = Extremely]. Then, participants were asked, “Do you think your opioid use increases your risk of harm from coronavirus (COVID-19)?” [0 = Definitely no to 7 = Definitely yes] [16]. Finally, respondents answered questions about opioid use and motivation to quit: “How has your opioid use changed since you learned about the coronavirus pandemic (COVID-19)?”, “How has your motivation to quit opioid use changed since you learned about the coronavirus pandemic (COVID-19)?” Each question allowed participants to select a nominal response option from: decreased, increased, stayed the same, other (please specify) [16].

##### Analytic plan

Prevalence values and means were calculated for background characteristics and COVID-19 related questions. Next, cross-tabulations between opioid use and motivation to quit were completed, followed by a logistic regression analysis to estimate the association between treatment as the outcome (yes/no) and opioid use (decreased/increased/stayed the same) and motivation to quit (decreased/increased/stayed the same) as the predictors of interest. An adjusted logistic regression model followed, which controlled for covariates (sex, age, race/ethnicity, education, and perception of whether opioid use increased risk of harm during COVID-19). In sensitivity analyses, for the ‘other’ category of opioid use and motivation to quit, 57 responses were persons who reported not using opioids since learning about COVID-19 (although within the past month). Therefore, the adjusted analyses were conducted after removing persons who specified they no longer used opioids amid the pandemic (n = 505). In other words, missing data was addressed with complete case analysis (listwise deletion). The model was checked for multicollinearity using the ‘collin’ command. All analyses were completed in Stata, version 16.

### Results

#### Descriptive outcomes: sociodemographics, opioid use and treatment, COVID-19 perceptions, opioid use and motivation to quit

**Fig. 1:**
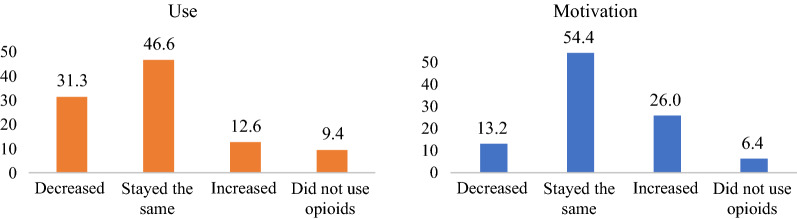
Opioid use and motivation to quit opioids since learning about COVID-19, (%). Approximately 94% participants who selected ‘Other’ for either question stated they did not use since learning about COVID-19; thus, this category has been labeled ‘Did not use opioids.’ Numbers may not sum to 100% due to rounding

The sample was 51% female, 67% Non-Hispanic White, 8% Non-Hispanic Black, and 15% Hispanic, with a median age of 34. Participants reported moderate concern about COVID-19 for their own health (mean 4.8; SD = 1.8), and for the health of others in their community (mean 5.3; SD = 1.6); they were ambivalent about the idea that their opioid use increased their risk of harm from COVID-19 (mean = 4.1, SD = 2.0). Approximately 22.1% (n = 124) of participants were actively enrolled in a treatment program for their opioid use. Similar proportions reported that their opioid use (46.6%) and motivation to quit opioid use (54.4%) stayed the same since learning about the coronavirus pandemic, whereas 31.3% of participants’ use decreased, and 26.0% of participants’ motivation to quit increased (Fig. 1). In examining the correlation between use and motivation, 37.5% of participants’ use and motivation stayed the same and 16.6% of individuals reported both that they decreased their use and their motivation to quit increased.

#### Regression outcomes: association between treatment, opioid use, and motivation to quit

In adjusted analyses, participants whose opioid use increased (AOR = 3.8; 95% CI 2.0, 7.5) and whose motivation to quit opioids decreased (AOR = 3.3; 95% CI 1.7, 6.3) were significantly more likely to be in treatment than persons whose use or motivation stayed the same, respectively. Persons who decreased their opioid use were significantly less likely to be in treatment than those whose use stayed the same (AOR = 0.5; 95% CI 0.2, 0.9; Table 1).

**Table 1:** Estimates from logistic regression with treatment as the outcome and opioid use and motivation to quit since learning about COVD-19 as the predictors of interest (n = 505)

	Odds ratio	95% confidence interval		AOR	95% confidence interval	
Opioid use						
Decreased	0.54	0.30	0.97	0.46	0.24	0.89
Increased	5.15	2.85	9.30	3.83	1.96	7.47
Stayed the same	1.00			1.00		
Motivation to quit						
Decreased	3.82	2.03	7.17	3.25	1.67	6.31
Increased	2.24	1.26	3.98	1.90	0.99	3.66
Stayed the same	1.00			1.00		
Male	–			0.51	0.30	0.85
Age	–			1.01	0.99	1.04
Race/ethnicity						
Non-Hispanic White	–			1.00		
Non-Hispanic Black	–			0.50	0.15	1.66
Hispanic	–			3.47	1.96	6.15
Non-Hispanic Other	–			0.96	0.38	2.40
Education						
Bachelor’s degree	–			1.00		
High school or less	–			1.21	0.61	2.40
Some college	–			0.35	0.17	0.72
Master’s degree or higher	–			1.13	0.57	2.25
Perception that opioid use increases harm from COVID-19	–			1.37	1.18	1.59

Adjusted model included sex, age, race/ethnicity, education, and opioid use harm perception. These estimates reflect analyses conducted after removing persons who reported no longer using opioids since learning about COVID-19 (n = 57)

### Discussion

#### Descriptive outcomes: sociodemographics, opioid use and treatment, COVID-19 perceptions, opioid use and motivation to quit

Participants in this study reported relatively moderate concerns about COVID-19 for their own health. While parallel research is limited, this conceptually mirrors other findings where such concerns tend to slightly exceed the scale midpoint, such as from a US college student validation of the Fear of COVID-19 scale [27], and data collected on perceived vulnerability to and severity of COVID-19 [28]. However, these perceptions are also subject to change given the rapidly-expanding knowledge base about COVID-19.

Fewer than one-quarter (22.1%) of participants reported being enrolled in a treatment program for prescription opioid or heroin use. Estimates of treatment utilization often vary depending on the way in which treatment is defined (e.g., outpatient office-based treatment, methadone, jail-based treatment, or group programs, among others). This study remained agnostic as to the type of treatment, except specifying the substances of focus. Analysis of data from a similar question among individuals ages 12 and older, administered via the 2005 to 2013 National Surveys on Drug Use and Health (NSDUH), identified a 19% prevalence of opioid-specific treatment among those with OUD [29]. However, the 2015 NSDUH identified marked differences between the percentage needing vs receiving specialty treatment for illicit drug use among ages 18 and older (19.6%) and 26 and older (24.1%) [30]. Given the median age of this sample (36 years) and the restriction to ages 18+, the prevalence of reported treatment engagement appears plausible, lending conceptual validity to the study.

Interestingly, the majority of individuals reported that their opioid use (46.6%) and/or motivation to quit (54.4%) remained the same since learning about COVID-19. Smaller percentages indicated that their use decreased (31.3%) and/or motivation to quit increased (26.0%) since learning about COVID-19. This stands in contrast to individuals’ responses about their tobacco use, where there was an increase in tobacco product use after learning about COVID-19 [16]. Although the survey results are cross-sectional and limited by the need to collect data rapidly in response to an evolving pandemic, researchers should conduct further, rigorous investigation into the degree to which the COVID-19 pandemic may represent not just a risk to persons who use opioids, but also a potential treatment leverage point where individuals may have heightened motivation to engage with provider systems.

Finally, roughly twice as many participants reported decreased (vs. increased) opioid use and increased (vs. decreased) motivation to quit, suggesting this may be an opportune time for clinical outreach and intervention among similar individuals. The subset of our sample that reported greater motivation to stop their opioid use and/or decreased opioid use after learning about COVID-19 represents an opportunity for innovative and ‘low threshold’ paths to treatment [10].

#### Regression outcomes: association between treatment, opioid use, and motivation to quit

The finding that increased opioid use was associated with reporting engagement with treatment, as was decreased motivation to quit, requires interpretation. Perception of harm from COVID-19 was only modestly associated with treatment engagement. An appropriate cross-sectional interpretation is that “participants who reported being in treatment were more likely to also be participants who reported increased use since learning about COVID-19.” These data were not collected temporally, so engagement with treatment may have been a consequence, rather than an antecedent, of increased use—or the converse. Decreased motivation (relative to motivation that stayed the same) for those engaged in treatment may reflect a broader, but unmeasured, multivariate relationship between treatment, substance use, and the pandemic.

While policy changes have been implemented to improve substance use treatment during COVID, access to treatment has remained limited [4]. US Drug Enforcement Agency (DEA) guidance allows exceptions during the pandemic-related emergency such as prescribing in states where providers are not registered with the DEA using telemedicine [31], but these options require individual provider adoption. The need for mutual aid gatherings (e.g., Narcotics Anonymous) to move online was identified early [32], but barriers to access and identification remained. These findings may also reflect more complex clinical phenotypes, the role of social isolation in opioid use and overdose [7], or associations between COVID-19 and poor mental health [33]. Decreased motivation to quit may likewise have resulted from changes to treatment procedures due to COVID-19 (e.g., shift to virtual visits), but this hypothesis would need to be tested through a separate study.

## Conclusion

The takeaway implication, as with the descriptive outcomes, is that there appears to be a shift in both risk and treatment access during the COVID-19 pandemic. At the same time, these data do not support the idea that this shift is driven directly by worry about COVID-19 itself. Rather, it is likely associated with a more complex interplay of causal factors. This brief data collection effort provides only a surface-level examination of issues, but these data indicate the need for further and deeper examination.

## Limitations

These cross-sectional data were collected as a rapid-response addendum to an extant study [17], so they provide only an indication of the areas on which researchers might profitably focus; the findings should not be generalized or used to influence policy or clinical practice without further study. In addition, this study analyzed several conceptually related but unidimensional constructs, while the impact of the pandemic likely affected numerous additional variables directly and indirectly, such as social isolation and alteration of substance access. Results should be understood as exploratory in light of this limitation. Conducting this study online via mTurk excluded adults who do not use the Internet, though this percentage was low as of 2019 (10%), with non-users primarily being ages 65 and older [34]. Further, key areas of difference between mTurk and the US population (age and education level) were controlled as covariates in the adjusted regression model [20–22].

## Data Availability

The datasets analyzed during the current study are available from the corresponding author on reasonable request.

## Abbreviations

AOR: Adjusted odds ratio
COVID-19: Coronavirus disease 2019
DEA: US Drug Enforcement Administration
HIT: Human Intelligence Task
mTurk: Amazon Mechanical Turk
NSDUH: National Survey on Drug Use and Health
OUD: Opioid use disorder
